# Aerobic Exercise Intervention, Cognitive Performance, and Brain Structure: Results from the Physical Influences on Brain in Aging (PHIBRA) Study

**DOI:** 10.3389/fnagi.2016.00336

**Published:** 2017-01-18

**Authors:** Lars S. Jonasson, Lars Nyberg, Arthur F. Kramer, Anders Lundquist, Katrine Riklund, Carl-Johan Boraxbekk

**Affiliations:** ^1^Department of Radiation Sciences, Diagnostic Radiology, Umeå UniversityUmeå, Sweden; ^2^Umeå Center for Functional Brain Imaging, Umeå UniversityUmeå, Sweden; ^3^Center for Demographic and Aging Research, Umeå UniversityUmeå, Sweden; ^4^Department of Integrative Medical Biology, Physiology, Umeå UniversityUmeå, Sweden; ^5^Departments of Psychology and Mechanical and Industrial Engineering, Northeastern UniversityBoston, MA, USA; ^6^Beckman Institute, University of Illinois at Urbana-ChampaignUrbana-Champaign, IL, USA; ^7^Department of Statistics, Umeå School of Business and Economics, Umeå UniversityUmeå, Sweden; ^8^Danish Research Centre for Magnetic Resonance, Copenhagen University HospitalHvidovre, Denmark

**Keywords:** aerobic exercise, cognition, executive function, plasticity, hippocampus, prefrontal cortex, freesurfer, transfer

## Abstract

Studies have shown that aerobic exercise has the potential to improve cognition and reduce brain atrophy in older adults. However, the literature is equivocal with regards to the specificity or generality of these effects. To this end, we report results on cognitive function and brain structure from a 6-month training intervention with 60 sedentary adults (64–78 years) randomized to either aerobic training or stretching and toning control training. Cognitive functions were assessed with a neuropsychological test battery in which cognitive constructs were measured using several different tests. Freesurfer was used to estimate cortical thickness in frontal regions and hippocampus volume. Results showed that aerobic exercisers, compared to controls, exhibited a broad, rather than specific, improvement in cognition as indexed by a higher “Cognitive score,” a composite including episodic memory, processing speed, updating, and executive function tasks (*p* = 0.01). There were no group differences in cortical thickness, but additional analyses revealed that aerobic fitness at baseline was specifically related to larger thickness in dorsolateral prefrontal cortex (dlPFC), and hippocampus volume was positively associated with increased aerobic fitness over time. Moreover, “Cognitive score” was related to dlPFC thickness at baseline, but changes in “Cognitive score” and dlPFC thickness were associated over time in the aerobic group only. However, aerobic fitness did not predict dlPFC change, despite the improvement in “Cognitive score” in aerobic exercisers. Our interpretation of these observations is that potential exercise-induced changes in thickness are slow, and may be undetectable within 6-months, in contrast to change in hippocampus volume which in fact was predicted by the change in aerobic fitness. To conclude, our results add to a growing literature suggesting that aerobic exercise has a broad influence on cognitive functioning, which may aid in explaining why studies focusing on a narrower range of functions have sometimes reported mixed results.

## Introduction

During the last two decades, numerous investigations have addressed the relationships between physical exercise and cognition (e.g., Khatri et al., 2001; Fabre et al., 2002; Legault et al., 2011; Langlois et al., 2013). Despite the wealth of literature showing how staying physically active may prevent cognitive decline (Yaffe et al., 2001; Sofi et al., 2011), dementia onset (Laurin et al., 2001; Yaffe et al., 2009), and improve brain-behavior relationship throughout the lifespan (Boraxbekk et al., 2016), there are also studies, e.g., a recent Cochrane review (Young et al., 2015), that have concluded that aerobic exercise, compared to active control training, had no added benefit on any cognitive function investigated. In contrast, several studies have shown that physical exercise positively influences a variety of cognitive processes, including controlled-processing (Chodzko-Zajko and Moore, 1994), processing speed (Dustman et al., 1994), executive control (Kramer et al., 1999), and visuospatial ability (Shay and Roth, 1992). The positive influence of physical exercise on cognition have also been supported by meta analyses (Colcombe and Kramer, 2003; Angevaren et al., 2008; Smith et al., 2011). Partially responsible for the somewhat equivocal results in the past may be that studies have translated results from an isolated task to reflect an effect on a cognitive domain (Blumenthal and Madden, 1988; Dietrich and Sparling, 2004; Oken et al., 2006), which has also been recognized by others (Roig et al., 2013). Some have even argued that to study plasticity related changes in cognition, several tasks tapping the same cognitive construct is critical for truly stating interventional effects (Noack et al., 2009). Another issue that may explain divergent findings is that designs, e.g., training protocols, and sample-specific characteristics, differ between studies (Etnier et al., 1997; Voelcker-Rehage and Niemann, 2013).

Parallel to examining cognitive functions in relation to exercise, studies have also started to examine the effects of exercise on brain structures; in particular in the hippocampus (Pereira et al., 2007; Erickson et al., 2011; Maass et al., 2014; Niemann et al., 2014; Thomas et al., 2016), and in frontal areas (Colcombe et al., 2006; Erickson et al., 2010; Flöel et al., 2010; Voss et al., 2013; Oberlin et al., 2016). One approach to study brain-behavior relationships stem from the underlying processes logic (Greenwood and Parasuraman, 2016), which implies that if there are observable structural changes in the brain from exercise, cognitive functions relying upon processing in those specific areas in the brain, may also benefit (Erickson et al., 2011; Voss et al., 2013; Burzynska et al., 2015). However, in the exercise literature mixed support for this hypothesis have been presented, with some studies failing to link exercise related differences in gray matter structure to cognitive changes (e.g., Colcombe et al., 2006; Burns et al., 2009; Ruscheweyh et al., 2011; Johnson et al., 2012), whereas others show a positive association between aerobic exercise, hippocampus gray matter morphology, and episodic memory performance (Erickson et al., 2009, 2011). Support for the association in regions of the brain besides the hippocampus is, however, scarce with two cross-sectional studies showing a link between aerobic fitness, executive functions, and prefrontal gray matter volume (Erickson et al., 2007; Weinstein et al., 2012, see also Voss et al., 2013; Oberlin et al., 2016 for frontal white matter structure, and Colcombe et al., 2004; Voelcker-Rehage et al., 2011, for cardiovascular responses in PFC and ACC, in relation to exercise-induced cognitive changes). Considering the general improvements on cognitive functions from aerobic exercise (Colcombe and Kramer, 2003; Angevaren et al., 2008). We decided to focus on three frontal regions that we predicted would undergo changes from exercise, and predict cognitive improvements according to the underlying processes logic. The dorsolateral prefrontal cortex (dlPFC) is involved in selecting and manipulating information in working memory whereas the ventrolateral PFC (vlPFC) is more involved in maintaining information in working memory (D'Esposito et al., 1999). The role of the anterior cingulate cortex (ACC) on the other hand is related to monitoring processes (Botvinick et al., 2004).

The aims of the present study were to investigate whether an aerobic exercise intervention (i) improves cognition in a general, non-construct-specific, sense, or specifically to certain cognitive constructs and (ii) alters cortical thickness or volume in brain structures important for cognition, specifically the dlPFC, vlPFC, ACC, and hippocampus.

## Materials and methods

### Participants

A total of 60 participants (64–78 years old) were recruited through an advertisement in the local newspaper. When applicants showed interest in participating in this study we contacted them to explain the purpose of the study as well as to verify that they were eligible to participate. Exclusion criteria included diabetes, medication known to influence dopamine, having a neurological disease, scoring below 27 on the mini-mental state examination (MMSE), having claustrophobia, or regularly performing moderately high to high-intensity exercise. A radiologist assessed structural images but no abnormality warranting exclusion was found. Participants provided written informed consent prior to the start of the study and were compensated with 1000 SEK for their participation. After baseline assessments, the participants were randomized into either an aerobic training group or an active stretching and toning control group. One female participant in the aerobic group had to discontinue training due to a foot injury, and one male participant in the control group due to a knee problem. Thus, the final sample included 29 participants in the aerobic training group and 29 participants in the active control (Table 1). The regional ethical committee in Umeå, Sweden, approved this study.

**Table 1:** **Sample characteristics**.

	**Aerobic**		**Control**		
	**N**	**Mean ± SD**	**N**	**Mean ± SD**	**Statistics**
Females (%)	29	52	29	59	$\chi_{\left(1\right)}^{2}$ = 0.279, *p* = 0.597
Age (years)	29	68.40 ± 2.54	29	68.97 ± 2.91	*t*_(55)_ = 0.789, *p* = 0.434
Education (years)	29	13.69 ± 3.49	29	13.69 ± 4.68	*t*_(52)_ = 0.191, *p* = 0.575
MMSE	29	28.96 ± 1.16	29	29.46 ± 0.64	*t*_(40)_ = 1.977, *p* = 0.055
Attendance (%)	29	85.13 ± 9.16	29	82.11 ± 10.43	*t*_(55)_ = 1.227, *p* = 0.225

### Procedure

After inclusion, each participant was scheduled for baseline data collection on six separate days. On day one, participants underwent aerobic fitness testing and body composition measurements (bone densitometry, not reported here). Neuropsychological testing was performed on three occasions on three separate days. On additional 2 days, scanning of the brain was made with magnetic resonance imaging (MRI, with T1 weighted imaging reported here, but also resting-state functional MRI, diffusion tensor imaging, anterior spin labeling, C2-C3 blood flow, and T2 weighted imaging), as well as positron emission tomography to measure dopamine D2 receptors (not reported here). In addition, several questionnaires about daily activities, sleep, motivation, subjective memory, and wellbeing were filled out but will not be reported here.

### Aerobic fitness

Aerobic fitness was estimated from a standardized graded cycle (Monark 839E, Monark Exercise AB, Sweden) ergometer test performed by an experienced tester at the Sport Science Lab at Umeå School of Sport Sciences. Every 3 min, the resistance was incremented by 30 W, with starting values being 30 W for females, and 40 W for males. Expired air was measured through a mouthpiece and analysis of O_2_ uptake (VO_2_ = O_2_ ml/kg ^*^min) was performed in an open system (Oxycon Pro, Erich Jaeger GmbH & Co, Würzburg, Germany) every 20 s. Heart rate was registered each minute with a Polar Sport chest transmitter (Polar H1, Kempele, Finland). Self-perceived exertion was assessed on a 15-point RPE-scale (ranging from 6 to 20) (Borg, 1970) at the end of each 3 min interval. The test was terminated at volitional exhaustion or when the self-perceived exertion was rated 15 or above at baseline, and 17 at follow-up. VO_2_ peak was estimated as the highest VO_2_ reached before test termination.

### Aerobic training

In the aerobic training group, participants trained in order to increase their VO_2_ by walking or jogging on an athletic indoor track, cycling on stationary cycles, or using cross-trainers. Their heart rate (HR) was monitored while they exercised 3 days a week for 6 months (30–60 min per session). As training continued, heart rate (HR) load of each session was increased incrementally, from 40 to 80% of their estimated maximum HR.

### Stretching and toning control condition

In the stretching and toning control condition, participants performed circuit training involving various bodily exercises aimed to improve muscle strength, flexibility, and balance without influencing VO_2_ to a large extent. They made between 6 and 10 repetitions per exercise and each exercise had various levels of difficulty.

### Neuropsychological test battery

**Figure 1:**
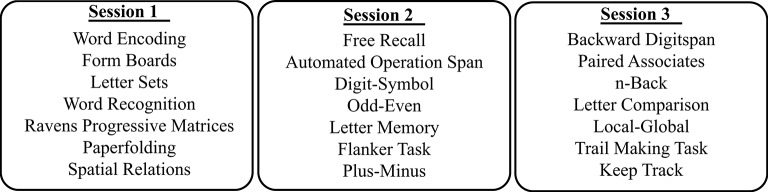
**Flow chart showing the neuropsychological test battery administration**.

Testing was performed on three separate days and lasted between 60 and 90 min per session. On the first day, tests of reasoning and visuospatial ability were performed. Trials on those tasks were self-paced and served to accommodate participants to the testing situation and computerized testing. On all computerized tasks recording reaction times (RT), individual responses deviating by more than 2.5 standard deviations from the mean were excluded. In addition, RT on a given trial was included only for correct responses. Unless stated otherwise, the tasks were presented using the E-Prime 2.0 software (Psychology Software Tools, Pittsburgh, PA). Each task will be described under its respective cognitive domain but see Figure 1 for a flow chart of the task order.

#### Episodic memory

##### Word recognition

Participants were required to encode a list of visually presented words for later retrieval (Nyberg et al., 1996). A list containing 30 nouns was studied in the encoding phase, 3 s per word with an interstimulus interval (ISI) of 1 s. Approximately 25 min later, a recognition phase commenced. Again, 30 words were presented, 4 s per word with an ISI of 1 s. Fifteen of the words were new, not from the original list presented during encoding, and 15 were words from the original list. With a key press, participants had to decide whether the word was presented in the original list or not. A practice block of five words was performed before the 30 nouns were studied. All words were different at baseline and post-intervention. The dependent measure was accuracy.

##### Free recall

In this task (Murdock, 1962), 16 Swedish nouns were visually presented, 3 s per word, with an ISI of 1 s. Immediately following the list presentation, participants were required, on a blank sheet, to write down as many of the 16 words as possible. Words were different at baseline and post-intervention. The dependent measure was the number of correctly recalled words.

##### Paired associates

In this task (Rohwer et al., 1967), word pairs comprised of Swedish nouns were studied. The word pairs were presented at a rate of 3 s per word pair, with an ISI of 500 ms. Two blocks of ten word pairs were performed. After each block, participants were presented with a sheet of paper where the first word in each word pair was printed. The task was to write down the word missing. Ten word pairs consisted of related nouns, e.g., head-nose, and the other ten of unrelated nouns, e.g., fork-flower. One practice block with five word pairs, three related and two unrelated, were performed before the two task blocks. Words were different at baseline and post-intervention. The dependent measure was the total number of correct words.

#### Processing speed

##### Trail making task 2 and 3

D-KEFS (Delis-Kaplan Executive Function System) (Delis et al., 2001) pen and paper trail making task (TMT) series 2 and 3 measure processing speed. In series 2, subjects were instructed to connect the numbers 1 through 16, as quickly as possible, by drawing a line with a pen between consecutive numbers. In series 3, the letters A through P were to be connected. Participants were also instructed that lines could not cross. The dependent measure was the sum of the times in seconds taken to complete the two series.

##### Digit-symbol task

In a computerized version of the Wechler Adult Intelligence Scale-Revised (WAIS-R), Digit-Symbol task, an array of nine digit-symbol combinations was shown on the upper half of a screen. Digits were always sorted in ascending order. On the lower half, a single digit-symbol combination was presented and the task was to, as quickly as possible, indicate with a key press whether the same digit-symbol combination could be found in the array. A single practice block consisting of ten trials with feedback was performed prior to three consecutive task blocks of 30 trials each. The dependent measure was RT.

##### Letter comparison

In this simple choice RT task, two lowercase letters were simultaneously presented. The task was to, as quickly as possible with a key press, indicate whether the two letters were equal or not. A 10 trial practice block with feedback was performed before two 30 s task blocks. The dependent measure was RT.

#### Executive function

##### Automated operation span

In the automated version of the operation span task (Unsworth et al., 2005), the goal was to memorize sequences of letters while judging simple mathematical operations true or false. These two tasks were practiced both separately and together before the real task begun. In four practice blocks, two and three letter sequences were displayed, 1 s per letter with an ISI of 250 ms. After each sequence, a response screen with 12 boxes containing a capitalized letter appeared. Using the mouse, the previously shown letters had to be selected in the correct order of appearance before the next block commenced. After four practice blocks of letter recall, 15 mathematical operations had to be solved. A mathematical operation [e.g., (9/3) + 2 = ?] was displayed in the center of the screen and participants were instructed to mentally solve the problem. When knowing the answer to the operation they were instructed to click the mouse to advance to the next screen. On the upper part of the screen a number appeared. If that number was the correct solution to the math problem (i.e., 5 in our example), a box stating “TRUE” had to be clicked, and if incorrect (e.g., 3), a box stating “FALSE.” After solving the 15 equations, participants practiced for three blocks on the real task with two letter sequences, performing both math operations and letter recall. After each math judgment, a letter was presented for 1 s. When the two math problems had been solved, and their corresponding letters had been presented, the response screen with 12 boxes appeared. After selecting the boxes with the remembered letters, the next block commenced. For the 10 task blocks (2 × 3–7 letter sequences), participants were instructed to maintain at least 85% accuracy on the math equations, which was indicated in the upper right corner of the screen. This was done to ensure that participants did not simply memorize letters, which was the outcome. After each block, feedback was presented, stating the math performance and how many letters were correctly remembered. The dependent measure was the sum of correctly remembered sets multiplied by the respective set size.

##### Flanker task

In the Flanker task (Eriksen and Eriksen, 1974), five arrows were presented in the center of the screen for 2 s, with an ISI of 2 s. These arrows were either congruent (e.g., < < < < <) or incongruent (e.g., < < > < <). The task was to focus on the direction of the middle arrow, and indicate with a left arrow press if the arrow pointed toward the left, and a right arrow press if pointing toward the right. On 50% of the trials, the arrows were congruent, and on the other 50% they were incongruent. After a 17 trial practice block, four blocks of 17 trials were performed. The dependent measure was the cost in RT on incongruent compared to congruent trials.

##### Backward digit span

In this computerized version of the WAIS-R digit span backward task, a sequence of presented numbers, 1–9, had to be memorized and responded to in backward order (answer to 5 9 2 is 2 9 5). Numbers were presented for 1 s, with an ISI of 250 ms, at the center of the screen. Pressing the corresponding keyboard numbers provided the response. Two sequences of two numbers with feedback served as practice. After practice, set length started with three numbers. If a correct response were given, the difficulty increased by one number, to a maximum of nine. If an incorrect response was given, the same sequence length was provided again. After two incorrect responses on a given sequence length the task was terminated. The dependent measure was the highest sequence length completed correctly.

#### Updating

##### Letter memory

In the Letter Memory task (Dahlin et al., 2008), a sequence of letters (A, B, C, or D) were pseudo-randomly displayed, 2 s per letter and with an ISI of 1 s. After the last letter was presented a response screen appeared and the task was to indicate the last four letters in the array by pressing the corresponding key. In other words, the task was not to remember the full length of the sequence but to consistently update working memory to hold the last four letters. Two practice blocks, 5 and 7 letter sequences, were performed with feedback, prior to 8 task blocks. The task blocks were 7, 9, 11, or 13 letter sequences that were performed twice. The dependent measure was accuracy.

##### N-back

In n-back (Kirchner, 1958) the task was to indicate with a key press within 2 s from stimulus onset, whether the digit presently on screen was the same digit as the digit presented 1 stimulus (1-back) or 2 stimuli (2-back) back. A 20-digit sequence was presented, 1.5 s per digit and an ISI of 500 ms. A practice 1-back block with feedback was performed prior to two 1-back task blocks. After 1-back, a practice 2-back block was performed prior to four task blocks. The dependent measure was 2-back accuracy.

##### Keep track

In the Keep Track task (Miyake et al., 2000), 15-word sequences were presented, 1.5 s per word with an ISI of 500 ms. Each word belonged to one of six categories: colors, metals, distances, countries, relatives, or animals. For each block, 2–4 target categories were displayed in boxes positioned in the lower part of the screen. The task was to recall the last seen word belonging to each of the target categories and write the answer on a sheet of paper. In two practice blocks with feedback, there were two target categories. In six subsequent task blocks, the number of target categories was either three or four. The dependent measure was the sum of correct blocks, times the number of target categories for each of those blocks.

#### Task-switching

##### Trail making task 4

D-KEFS pen and paper trail making task (TMT) series 4 was used to measure task-switching ability. In series 4, subjects were instructed to, as quickly as possible, connect the numbers 1 through 16 and the letters A through P by drawing a line with a pen between consecutive numbers/letters. The task was to begin with the number 1, connecting 1 to letter A, from A to 2, from 2 to B, and so on. Participants were also instructed that lines could not cross. The dependent measure was the additional time taken to complete TMT 4 compared to completing TMT 2 and 3, i.e., the switching cost.

##### Odd-even

A cued task-switching paradigm was used (Rogers and Monsell, 1995; Jonasson et al., 2014). The target consisted of a letter-digit pair presented a few centimeters apart. On each trial, the sequence of events was as follows: fixation cross (1000 ms), task cue (250 ms), cue-target interval (1500 ms), and target (2000 ms). Task cues indicated the upcoming task, “Attend Letter” or “Attend Digit,” the former indicating a consonant-vowel judgment, and the latter an odd-even judgment. The task cues were randomly alternated, such that there were a task switch on 50% of the trials, and a task-repeat on the other 50%. A practice block of 20 trials was completed with feedback, followed by three task blocks without feedback. Odd and consonant judgments were recorded with a left-arrow press, whereas even and vowel judgments were recorded with a right-arrow press. The dependent measure was the cost in accuracy between switch and no-switch trials.

##### Local-global

In this task, Navon figures were used (Navon, 1977), where a large figure (global level), is comprised of smaller figures (local level). The larger figure was always a circle or a triangle, comprised of smaller triangles or circles, respectively, such that both circle and triangle were represented either as a global object or as local objects. These were shown on screen for 3 s, with an ISI of 1 s. If the color of the figure was blue, the task was to judge whether the global figure was a circle (right button), or a triangle (left button). If the color of the figure was black, the task was to judge whether the local objects were circles (right button), or triangles (left button). On 50% of the trials the same rule was repeated, and on the other 50% a rule switch was required. A 20 trial practice block was first performed with feedback, followed by three task blocks. The dependent measure was the cost in accuracy between switch and no-switch trials.

#### Reasoning

##### Letter sets

On this reasoning task (Salthouse, 2005; Baniqued et al., 2013), the goal was to identify which of five alternative letter sets were odd. Four of the sequences shared some common rule not shared by the fifth, odd, letter set. Two practice problems were explained before the task commenced. Participants were instructed that they had 10 min to complete as many of the letter sets as possible. The dependent measure was the number of accurate trials, corrected for guessing.

##### Ravens progressive matrices

In this version of Ravens progressive matrices (Salthouse, 2005; Baniqued et al., 2013), a 3 by 3 array with patterns was presented. The pattern in the lower right corner was missing, and from eight alternatives, the pattern that will complete the array could be found. After two practice trials with feedback, 10 min were provided to complete as many trials as possible, for a maximum of 18. A response was given by pressing the corresponding alternative's number on a keyboard. The dependent measure was accuracy, corrected for guessing.

#### Visuospatial ability

##### Form boards

In form boards (Salthouse, 2005; Baniqued et al., 2013) the task was to produce a figure by combining 2–5 alternative pieces, similar to a puzzle. The target figure was displayed on the upper part of the screen, and the five pieces on the lower part of the screen. After having decided which non-overlapping pieces were required to produce the figure, each piece was selected with a mouse click. Participants were instructed on a practice trial, before they were given 8 min to complete as many figures as possible. The dependent measure was the number of accurate figures completed.

##### Paper folding

In this task (Salthouse, 2005; Baniqued et al., 2013), participants could see images of a square paper being folded in the upper part of the screen. After 3–4 folds, a number of holes were punched through all layers of the folding. The task was to mentally unfold the paper, and select the correct image showing the location of the holes. There were five alternatives and the response was given by clicking with the mouse on one of the five images. After a practice trial, 10 min were given to complete as many trials as possible, to a maximum of 12. The dependent measure was the number of accurate trials, corrected for guessing.

##### Spatial relations

In this task (Salthouse, 2005; Baniqued et al., 2013), a target figure was displayed on the upper part of the screen. The task was to select the figure, from five alternatives displayed in the lower part of the screen, which could be rotated to fit the target figure. After two practice trials, 10 min were given to complete as many trials as possible, to a maximum of 20. The dependent measure was the number of accurate trials, corrected for guessing.

#### Failed task

##### Plus-minus

The goal of this task was to measure task-switching ability. Participants were presented with three sheets of paper on which the answers should be written, each with two columns of numbers ranging from 1 to 100. On the first sheet, the task was to add three to each number, on the second sheet, subtract three, and on the final sheet, alternate between adding three and subtracting three. Unfortunately, a considerable number of participants had problems writing numbers with a pencil within a reasonable time frame and therefore the task was removed from the analyses.

### Magnetic resonance imaging acquisition

Structural imaging was performed on a 3T General Electric scanner equipped with a 32-channel head coil. High-resolution T1-weighted structural images were collected with a 3D fast spoiled gradient echo sequence (180 slices with a 1 mm thickness, TR 8.2 ms, TE 3.2 ms, flip angle 12°, field of view 25 × 25 cm).

### Brain segmentation

Freesurfer (Fischl et al., 2002), version 6-beta (20151015) longitudinal stream (Reuter et al., 2012), was used to segment the brain into known anatomical structures. In the longitudinal stream a subject specific base template is created from several time points. Errors visually observed on the base template were manually edited prior to creation of the final longitudinal segmentation. For the subcortical segmentations, the volume (mm^3^) of hippocampus was used as dependent measures. For the cortical segmentation, the dependent measure was cortical thicknesses (mm) from the Destrieux atlas (Destrieux et al., 2010).

Similar to Vijayakumar et al. (2014), a dlPFC region-of-interest (ROI) was produced by combining the bilateral superior and middle frontal gyri, a vlPFC ROI by combining the bilateral opercular, orbital, and triangular gyri, and an ACC ROI by combining the bilateral anterior and middle-anterior part of the cingulate gyri and sulci.

### Statistical analyses

#### Neuropsychological test battery

Each cognitive construct, episodic memory (EM), processing speed (PS), updating (UPD), task-switching (TS), executive function (EF), and visuospatial ability (SRS) constructs included three different tasks. The reasoning construct (RS) included two tasks. The cognitive tasks were first z-transformed and averaged to form the unit-weighted EM, PS, UPD, TS, EF, RS, and SRS constructs. Missing values were imputed based on the score on the completed tasks belonging to the same construct. For any participant, not more than one task score for any given construct was missing.

#### Confirmatory factor analysis

To confirm the underlying factor structure of the cognitive battery, confirmatory factor analyses (CFA) were performed with the lavaan package in R (http://CRAN.R-project.org/package=lavaan). CFAs were made with the EM, PS, UPD, TS, and EF tasks. Due to the small sample size, we considered a unit-weighted “Cognitive score” to be more reliable than the latent score of a general higher-level factor (Wolf et al., 2013), hence the “Cognitive score” was computed by averaging the constructs used in the CFA. The RS and SRS constructs involved self-paced tasks related to reasoning or problem-solving ability and were thus analyzed in two separate CFAs, one with a single factor and one with two factors. See Supplementary 1 for further CFA details.

#### Group differences

Two-way repeated measures analysis of covariance (rmANCOVA) was performed to compare within and between groups differences in aerobic fitness. Two separate two-way repeated measures multivariate ANOVAs (MANOVA) were performed on the cognitive constructs from the CFA, and on the Freesurfer segmentations; dlPFC, vlPFC, ACC cortical thickness, and hippocampus volume. All analyses were conducted with age, gender, and education as covariates, and, unless stated otherwise, significant tests were also significant without the addition of covariates.

#### Associations of aerobic fitness, cortical thickness, and cognitive performance

To understand the specific influence of aerobic fitness on cortical thickness, hippocampus volume, and “Cognitive score,” additional analyses were performed using linear regressions both at baseline and with changes over time. The regressions are reported with the covariates age, gender and education regressed out. One participant was excluded due to having an aerobic fitness > 3 SD above the mean. For the regressions involving cortical thickness and hippocampus volume, the freesurfer derived intracranial volume was regressed out. To analyze changes over time, delta scores were computed by subtracting baseline scores from post-intervention scores.

In order to understand the connection between aerobic fitness, cortical thickness, hippocampus volume, and cognitive performance, it is important also to examine the interplay between brain structure and cognitive performance. To that end, cortical thickness in dlPFC, vlPFC, and ACC were used as predictors for “Cognitive score.” Due to the well-known influence of the EF measures on dlPFC, vlPFC, and ACC, we performed additional regressions as control analyses. Similarly, EM was specifically tested in relation to hippocampus volume.

## Results

### Confirmatory factor analysis

Details for the CFAs are reported in Supplementary 1. In short, a five factor solution with a “Cognitive score” factor with loadings from EM, PS, UPD, and EF showed best fit, $\chi_{\left(50,N=118\right)}^{2}$ = 75.199, *p* < 0.012, RMSEA = 0.065, CFI = 0.946, AIC = 3652.311, SRMR = 0.079. The loading from UPD to “Cognitive score” was highest, followed by EF, PS, and finally EM. Due to the small sample size we do not consider the estimated loadings to be reliable however [see e.g., (Wolf et al., 2013) who, based on simulation studies, recommend *n* > 150 when all (true) loadings are expected to be above.8]. Hence, instead of using a factor score, the EM, PS, UPD, and EF constructs were averaged to form a unit-weighted “Cognitive score.”

### Group differences

**Figure 2:**
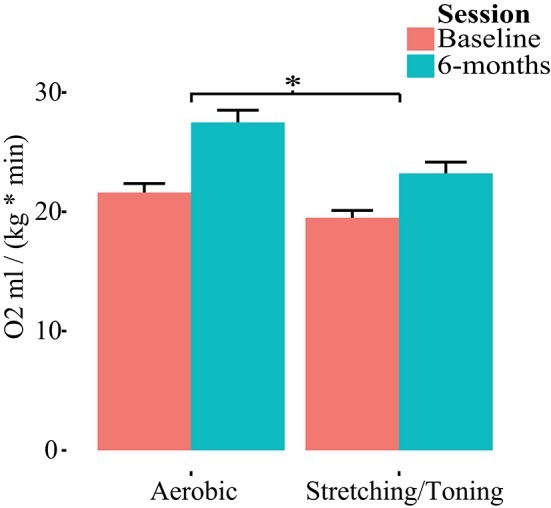
Group by time differences in aerobic fitness (O_**2**_ml/kg^*^min). Repeated measures of covariance, controlling for age, gender, and education revealed a group by time interaction favoring aerobic exercise. ^*^p < 0.05.

Both groups had, compared to baseline, higher aerobic fitness after the intervention (Table 2, Figure 2). The rmANCOVA revealed a significant group by time interaction favoring the aerobic group, *F*_(1, 53)_ = 5.215, *p* = 0.0264, confirming the desired effect of the intervention.

**Table 2:** **Group differences between aerobic exercise (***n*** = 29) and stretching/toning (***n*** = 29) in cognitive performance and aerobic fitness**.

	**Aerobic**		**Control**		**Change**	
	**Baseline Mean ± SD**	**6-months Mean ± SD**	**Baseline Mean ± SD**	**6-months Mean ± SD**	**Aerobic δ_RM_**	**Control δ_RM_**
Episodic Memory	−0.17 ± 0.50	0.08 ± 0.86	0.22 ± 0.87	0.17 ± 1.02	0.41	−0.08
Processing Speed	0.08 ± 0.61	0.37 ± 0.47	−0.12 ± 0.99	0.05 ± 1.04	0.76	0.40
Updating	−0.09 ± 0.49	0.10 ± 0.51	0.06 ± 0.61	0.12 ± 0.72	0.42	0.14
Executive Function	0.07 ± 0.71	0.50 ± 0.76	−0.06 ± 0.89	0.16 ± 0.91	0.79	0.45
Cognitive Score	−0.03 ± 0.42	0.26 ± 0.50	0.02 ± 0.62	0.13 ± 0.67	1.02	0.43
Aerobic fitness	21.60 ± 4.13	27.69 ± 5.53	19.50 ± 3.31	23.22 ± 5.06	1.73	1.20

Cognitive constructs and “Cognitive score” are reported as z scores and aerobic fitness as VO_2_ peak (O_2_ml/kg^*^min). In addition, effect size δ_RM_ estimates for each test and group were calculated. The formula provided by Morris and DeShon (2002), Equation 8, was used as it takes into account the test correlation between time points when calculating the effect size for repeated measures.

**Figure 3:**
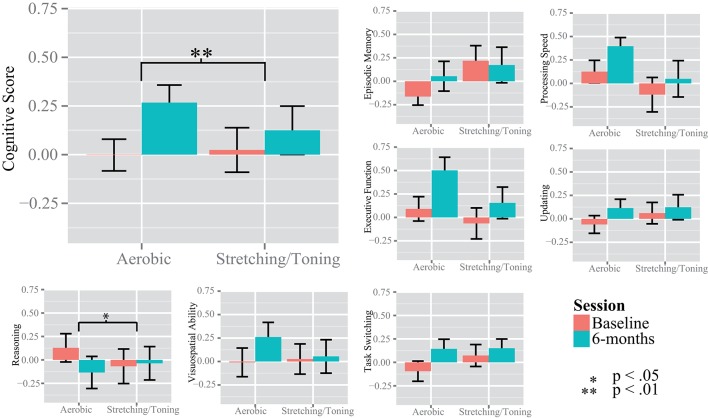
**Changes in cognition over time**. The y-axis show the standardized z value for the unit-weighted cognitive constructs. The “Cognitive Score” was derived from the episodic memory, processing speed, updating, and executive function tasks. Significance bars are derived from repeated measures analysis of variance, within and between groups.

Due to presence of six multivariate outliers we decided to conduct rmANCOVAs on the “Cognitive score” and the separate cognitive constructs instead of a MANOVA on the constructs (Table 2, Figure 3). A significant group by time interaction on the “Cognitive score” favored the aerobic group, *F*_(1, 53)_ = 7.700, *p* = 0.0076. There were no group by time interactions on EM, *F*_(1, 53)_ = 2.707, *p* = 0.1058, PS, *F*_(1, 53)_ = 2.090, *p* = 0.1542, UPD, *F*_(1, 53)_ = 1.411, *p* = 0.2402, and EF, *F*_(1, 53)_ = 2.080, *p* = 0.1551, or SRS, *F*_(1, 53)_ = 2.439, *p* = 0.1243. However, a significant interaction favoring the control group was seen on the RS construct, *F*_(1, 53)_ = 5.219, *p* = 0.0264, but only after controlling for age, gender, and education. For descriptive purposes, results for all tasks and constructs in the neuropsychological test battery, including within group rmANCOVAs, are reported in Supplementary Table 1.

The MANOVA with dlPFC, vlPFC, and ACC thickness, and hippocampus volume revealed no group differences in cortical thickness or hippocampus volume, *F*_(4, 108)_ = 0.128, *p* = 0.972; Wilks' Λ = 0.995. There were no group interactions or significant effects of time in any of the regions, all *p* > 0.05 (Table 3).

**Table 3:** **Cortical thickness and hippocampus volume per group and session**.

		**Aerobic Exercise**		**Control**		
	**N**	**Baseline Mean ± SD**	**6-months Mean ± SD**	**Baseline Mean ± SD**	**6-months Mean ± SD**	**Statistic *F*-test^a^**
dlPFC	29	2.59 ± 0.12	2.58 ± 0.11	2.60 ± 0.12	2.60 ± 0.12	*F*_(1, 53)_ = 0.002, *p* = 0.964
vlPFC	29	2.55 ± 0.11	2.53 ± 0.11	2.57 ± 0.11	2.57 ± 0.13	*F*_(1, 53)_ = 1.139, *p* = 0.291
ACC	29	2.59 ± 0.13	2.56 ± 0.15	2.57 ± 0.09	2.56 ± 0.11	*F*_(1, 53)_ = 0.763, *p* = 0.386
HPC	29	7462 ± 832	7430 ± 827	7683 ± 651	7668 ± 700	*F*_(1, 53)_ = 0.095, *p* = 0.760

a *Two-way repeated measures analysis of covariance with group and session, controlling for age, gender, and education. dlPFC, dorsolateral prefrontal cortex (mm); vlPFC, ventrolateral prefrontal cortex (mm); ACC, anterior cingulate cortex (mm); HPC, hippocampus (mm^3^)*.

### Associations of aerobic fitness, cortical thickness, and cognitive performance

**Figure 4:**
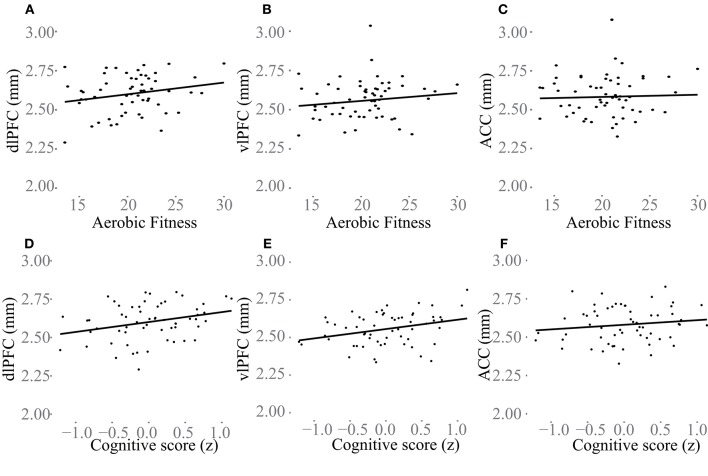
Baseline associations between aerobic fitness (O_**2**_ml/kg^*^min), “Cognitive score” (z) and cortical thickness (mm) in dlPFC (A,D), vlPFC **(B,E)**, and ACC **(C,F)**. In **(A–C)** aerobic fitness is plotted against cortical thickness, and in **(D–F)** “Cognitive score” is plotted against cortical thickness.

**Figure 5:**
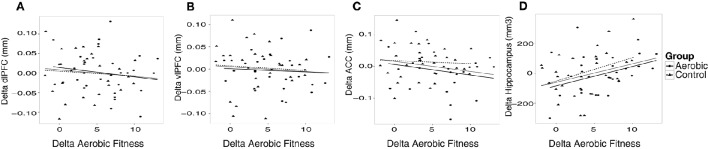
The change in cortical thickness and hippocampus volume plotted against change in aerobic fitness (O_**2**_ml/kg^*^min). Increases over time in cortical thickness (mm), hippocampus volume (mm^3^) and aerobic fitness have positive values. Change in aerobic fitness is plotted against change in thickness in **(A)** dlPFC, **(B)** vlPFC, **(C)** ACC, and **(D)** hippocampus volume. Observations from the aerobic group are circles, and the regression line is the thinner line. Observations from the control group are triangles, and the regression line is dashed. The thicker regression line is for the full sample.

Additional analyses were conducted to understand possible associations between aerobic capacity, cortical thickness, and cognitive performance. Results of the regressions are reported in Table 4. At baseline, higher VO_2_ peak was positively associated with dlPFC thickness, but was unrelated to vlPFC and ACC thickness as well as hippocampus volume (Figure 4). The change in VO_2_ peak was positively associated with the change in hippocampus volume, but unrelated to change in cortical thickness (Figure 5).

**Table 4:** **Associations between cortical thickness, aerobic fitness, and “Cognitive score”**.

	**Change**	**Aerobic Fitness^a^**		**“Cognitive score”^b^**	
	**Mean ± SD**	**Cross-sectional**	**Longitudinal**	**Cross-sectional**	**Longitudinal**
dlPFC	−0.01 ± 0.05	*F*_(1, 56)_ = 4.94, *p* = **0.03**, *r* = 0.28	*F*_(1, 55)_ = 1.12, *p* = 0.29, r = −0.14	*F*_(1, 57)_ = 6.75, *p* = **0.01**, r = 0.33	*F*_(1, 56)_ = 4.76, *p* = **0.03**, r = 0.28
vlPFC	−0.01 ± 0.05	*F*_(1, 56)_ = 3.07, *p* = 0.09, *r* = 0.23	*F*_(1, 55)_ = 1.52, *p* = 0.22, r = −0.16	*F*_(1, 57)_ = 6.82, *p* = **0.01**, r = 0.33	*F*_(1, 55)_ = 0.15, *p* = 0.70, r = 0.05
ACC	−0.02 ± 0.07	*F*_(1, 56)_ = 0.90, *p* = 0.35, *r* = 0.13	*F*_(1, 55)_ = 2.05, *p* = 0.16, r = −0.19	*F*_(1, 57)_ = 2.17, *p* = 0.15, r = 0.19	*F*_(1, 55)_ = 0.37, *p* = 0.55, r = 0.08
HPC	23.67 ± 140	*F*_(1, 56)_ = 0.49, *p* = 0.49, r = −0.09	*F*_(1, 55)_ = 9.68, *p* = **0.002**, r = 0.39	*F*_(1, 57)_ = 1.14, *p* = 0.29, r = 0.14	*F*_(1, 56)_ = 2.61, *p* = 0.11, r = −0.21

Linear regressions with cortical thickness (mm) and hippocampus volume (mm^3^) predicted by

a aerobic fitness, and

b *”Cognitive score,” controlling for age, gender, education, and intracranial volume. One outlier in baseline aerobic fitness and one outlier in cortical thickness change were removed. Associations were only considered significant (bold p-value) if significant both with and without covariates. dlPFC, dorsolateral prefrontal cortex; vlPFC, ventrolateral prefrontal cortex; ACC, anterior cingulate cortex; HPC, hippocampus*.

**Figure 6:**
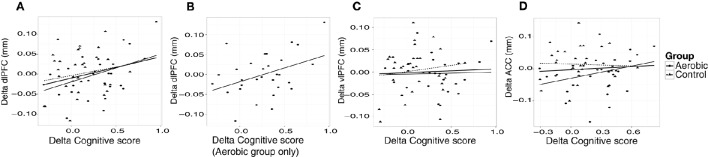
**Associations between changes in “Cognitive score” (z) and cortical thickness (mm)**. The change in “Cognitive score” is plotted against the change in thickness in, **(A)** dlPFC, **(B)** dlPFC for the aerobic group specifically, **(C)** vlPFC, and **(D)** ACC. Observations from the aerobic group are circles, and the regression line is the thinner line. Observations from the control group are triangles, and the regression line is dashed. The thicker regression line is for the full sample.

“Cognitive score” at baseline was significantly associated with larger thickness in dlPFC (*p* = 0.01) and vlPFC (*p* = 0.01), but not to ACC, or to hippocampus volume (Figure 4). Following the 6-month training period the change in “Cognitive score” was positively associated with the change in dlPFC (Figure 6). The association between changes in “Cognitive score” and dlPFC thickness was driven by the aerobic group, showing a significant effect, *F*_(1, 28)_ = 4.828, *p* = 0.037, *r* = 0.39, (Figure 6B) whereas the control group did not, *p* = 0.34.

Further analyses revealed construct specific influences in expected anatomical structures. Higher EF at baseline was specifically associated with thickness in dlPFC, *F*_(1, 57)_ = 8.167, *p* = 0.006, *r* = 0.35, vlPFC, *F*_(1, 57)_ = 5.596, *p* = 0.021, *r* = 0.30, and ACC, *F*_(1, 57)_ = 8.309, *p* = 0.006, *r* = 0.36, but not to hippocampus volume *F*_(1, 57)_ = 0.62, *p* = 0.44, *r* = 0.10. Conversely, EM showed a specific association with larger hippocampus volume at baseline, *F*_(1, 57)_ = 4.661, *p* = 0.035, *r* = 0.28, but not to cortical thickness. Over time, improved EF was positively associated with the change in dlPFC thickness specifically, *F*_(1, 57)_ = 4.761, *p* = 0.033, *r* = 0.28. EM was unrelated to changes in cortical thickness and hippocampus volume.

## Discussion

The aims of the present study were to investigate the influence of aerobic exercise on cognitive performance, and brain structures derived using Freesurfer. We observed that sedentary older adults randomly assigned to aerobic exercise exhibited a broad improvement in cognitive performance, reflected by a cognitive score, compared to individuals assigned to stretching and toning control training. Regarding brain structures, the results were rather equivocal with no direct effect of the intervention, but with aerobic fitness predicting cortical thickness in PFC at baseline, but not over time, whereas hippocampus volume exhibited the opposite pattern, with no association at baseline, but a positive association over the 6-month training period.

### Cognition

A considerable strength of the present investigation was the inclusion of several tests for each cognitive construct, providing the opportunity to investigate cognition in a broad sense. The CFA displayed good fit indices despite the small sample size, disregarding TS. Previous studies have generally had fewer tasks and/or investigated fewer constructs (Burns et al., 2009; Erickson et al., 2011; Legault et al., 2011; Thomas et al., 2016). Thus, the present observation that aerobic exercise compared to stretching and toning control training was more effective in improving cognition in older adults is an important contribution to a field showing equivocal results, owing at least in part, to a lack of robust test batteries enabling use of latent constructs. Different tasks have been used across studies and although the tasks are taken to represent the same cognitive constructs, this is not always the case, e.g., improved digit-symbol task performance in aerobically fit individuals could be taken to support the visuospatial hypothesis (Stones and Kozma, 1989), although this particular task is commonly viewed as a task measuring speed of processing (Salthouse, 1996). Thus, we support a general improvement in cognitive function which is in contrast to Young et al. (2015) but according to several other meta-analyses (Colcombe and Kramer, 2003; Angevaren et al., 2008; Smith et al., 2011). Our conclusion of an effect in a broad sense was further supported by our construct specific analyses. Compared to baseline, aerobic exercise resulted in improvements in several cognitive constructs; PS, UPD, EF, and SRS, with a trend also for EM (Figure 3, and Supplementary Table 1). The largest effect size was seen for EF, assimilating conclusions from an earlier meta-analysis (Colcombe and Kramer, 2003). The control group improved PS and EF abilities, albeit to a lesser degree, and not in the other cognitive constructs. This suggests that even low-intensity exercises aiming to promote strength, balance, and flexibility may have positive effects on cognitive functioning. Motor fitness and resistance training have been related to improved executive functioning (Liu-Ambrose et al., 2008; Voelcker-Rehage et al., 2010, 2011) and processing speed (Voelcker-Rehage et al., 2010, 2011), hence, group comparisons may mask aerobic exercise-induced changes to cognition due to training-induced improvements also in the control group. There was an interaction favoring the control group on the reasoning construct not expected a priori. However, there could have been a regression to the mean effect influencing this particular analysis (Figure 3), and the interaction was due to the aerobic group's worsened performance, rather than improved performance for the control group. In sum, our observations indicate that aerobic exercise, compared to stretching and toning control training improves cognition generally, rather than being specific to any given component.

### Prefrontal cortical thickness

Regarding aerobic exercise, fitness, and cortical thickness, results were rather equivocal. A direct effect of the intervention on cortical thickness in dlPFC, vlPFC, or ACC was absent. However, subsequent analyses indicated that aerobic fitness at baseline had a positive relationship with cortical thickness in dlPFC, partly consistent with previous cross-sectional data showing positive effects on PFC and ACC gray matter from aerobic fitness (Colcombe et al., 2003; Weinstein et al., 2012) and physical activity (Flöel et al., 2010). As the aerobic exercise group improved their aerobic fitness considerably compared to the control group one could have expected to find an effect from aerobic fitness in dlPFC over time, explaining also the improved cognitive performance, considering that “Cognitive score” predicted dlPFC thickness both at baseline and change over time in the aerobic group. However, one explanation is that the exercise-induced improvements in cognitive performance immediately after a period of training is not mainly due to gray matter changes, but may depend on altered cardiovascular responses (Colcombe et al., 2004; Voelcker-Rehage et al., 2011), white matter changes (Voss et al., 2013; Oberlin et al., 2016), functional connectivity (Voss et al., 2010, 2016), brain-derived neurotrophic factor (Leckie et al., 2014), or other molecular processes. Wheel-running for example, has been associated with alterations in dopaminergic markers in striatum (MacRae et al., 1987; Meeusen et al., 1997; Kim et al., 2016), and considering the influence of dopamine both on prefrontal and striatal function in relation to executive control functions, (Frank and O'Reilly, 2006; Cools and D'Esposito, 2011; D'Esposito and Postle, 2015) this could be a potential pathway for exercise-induced improvements in cognition.

Moreover, training duration may also be an important factor, where effects of exercise may be absent at 6-months, only to be revealed after 12 months (Voss et al., 2010). Duration could potentially explain why baseline levels, and not 6-month change, predicted cortical thickness, assuming that baseline fitness levels can be taken to represent an average fitness level, stretching further back in time than 6 months. It should also be noted that recent studies have shown that resistance training may also influence the brain, e.g., by altering cardiovascular responses in PFC and ACC (Voelcker-Rehage et al., 2011). The active control group likely improved both their motor fitness and muscle strength; hence, effects may be influenced also by factors we did not measure, and mask potential group comparisons. Future studies could include additional tests to control for changes in motor and muscle fitness. In sum, our analyses of cortical thickness are only partly consistent with the view that being aerobically fit at an older age preserves brain health in executive control regions (Kramer and Erickson, 2007; Prakash et al., 2015).

### Hippocampus volume

As opposed to the pattern observed in dlPFC, hippocampus volume did not show an association to aerobic fitness at baseline, as was presented by Erickson et al. (2009). Conversely, the change in aerobic fitness was positively associated with the change in hippocampus volume, replicating previous studies (Erickson et al., 2011; Thomas et al., 2016). This effect could be explained by increased neurogenesis (van Praag et al., 1999; van Praag, 2005) or angiogenesis (Pereira et al., 2007; Maass et al., 2014), as a function of aerobic fitness improvement. It should also be noted that resistance training has been linked to increased hippocampus volume (Niemann et al., 2014). Functionally, hippocampus volume was related to EM at baseline, but the change in hippocampus volume could not predict changes in EM. One explanation as to why hippocampus volume may not have been predictive of verbal EM over time is that new neurons may not have been incorporated in functional circuitry, considering that a large proportion of newborn hippocampal cells do not survive the first few weeks without environmental challenges (van Praag et al., 1999). An alternative explanation is that we used verbal memory tasks, and previous exercise studies finding a relation between EM and hippocampus used spatial memory tasks (Erickson et al., 2011; Maass et al., 2014), also in rodents (van Praag, 2005; Yau et al., 2012). Others, using verbal EM measures have also failed to find an association (Pereira et al., 2007; Thomas et al., 2016). Hence, spatial memory tasks could be more sensitive to structural changes in hippocampus (Bonner-Jackson et al., 2015).

### Limitations

Despite using the gold standard for estimating VO_2_ peak, we are limited in making firm claims concerning the absolute changes in VO_2_ peak. We speculate that this may be due mainly to two factors. Firstly, different termination thresholds were used at baseline and at follow-up. Secondly, leg strength has an influence on estimated VO_2_ peak when using a cycle ergometer test, (Diesel et al., 1990), and both training protocols were likely to influence leg strength. Including additional measurements on leg strength could have been informative and should be considered. In addition, including accelerometers or activity bracelets during the intervention period for monitoring everyday physical activity should be an important source of information for future studies.

Limitations of this study include only measuring two time points, 6 months apart. For instance, it appears as if hippocampus is sensitive to the timing of measurements, which was recently showed in a study observing that aerobic exercise may increase hippocampus volume within 6 weeks, while returning back to baseline values also within 6 weeks (Thomas et al., 2016). The time between aerobic fitness measurements and MRI acquisition in the present study was between 1 and 2 weeks, potentially introducing a source of noise. The issue of timing is also related to the lack of a clear understanding of the dose-response relationship between aerobic fitness and various brain indices (Prakash et al., 2015). For instance, Voss et al. (2010) found effects on functional connectivity in the default mode network and a frontal executive network only after 12 months, not after 6, suggesting that longer training or time between scans may be required to detect cortical changes.

Moreover, practice effects from repeated testing are a potential limitation affecting the cognitive results, but unlikely explain the improvements entirely despite the lack of interactions on specific constructs; due to the long test-retest duration, comparably large effect sizes (Makizako et al., 2013; de Oliveira et al., 2014; Goldberg et al., 2015), and an active control group that may also have exhibited training-induced cognitive improvements (Liu-Ambrose et al., 2008; Voelcker-Rehage et al., 2010). Finally, although white matter has also been investigated in relation to physical activity (Voss et al., 2013; Hayes et al., 2015; Oberlin et al., 2016) we restricted this study to mainly gray matter thickness in frontal regions and hippocampus volume. On that note, Freesurfer may also be less specific than voxel-based morphometry, or diffusion weighted imaging, as Freesurfer assigns voxels to either gray or white matter, thereby limiting our ability to be specific about the exact changes captured in our ROIs. Nevertheless, cortical thickness from Freesurfer has shown to be predicitive of cognitive functioning in older adults (Burzynska et al., 2012), and concords with voxel-based morphometry when assessing atrophy (Lehmann et al., 2011).

## Conclusion

In this study we conclude that aerobic exercise in sedentary older adults has the potential to improve cognition in a broad, rather than specific, sense, as captured in our “Cognitive score” based on episodic memory, processing speed, working memory updating, and executive function tasks. These results add to a growing literature suggesting that aerobic exercise has a broad influence on cognitive functioning, which may aid in explaining why studies focusing on a narrower range of functions have sometimes reported mixed results. In addition, dlPFC may be a key region of interest in frontal cortex for explaining exercise-induced effects on cognition, although larger studies are warranted. Cortical thickness in dlPFC predicted “Cognitive score” at baseline, as well as improvement in cognitive performance over time in the aerobic group only. That aerobic fitness is specifically related to increased cortical thickness or reduced cortical thinning of dlPFC in older adults was only given partial support however, with fitness showing a positive relation to dlPFC at baseline, but not over 6 months, despite the potential links to dlPFC and aerobic exercise via improved “Cognitive score.”

## Author contributions

LJ, LN, AK, KR, and CB designed the study; LJ and AL performed the statistical analyses; all authors contributed in revising the work and approved the final version of the manuscript.

## Funding

Support was obtained from the Swedish Research Council (2012-00530), Västerbotten County Council and Umeå University, the Swedish Research Council for Sport Science and Umeå School of Sport Sciences to CB, and from the Kamprad Family Foundation to LN.

### Conflict of interest statement

The authors declare that the research was conducted in the absence of any commercial or financial relationships that could be construed as a potential conflict of interest.
