# Supplementary material for: Aerobic Exercise Intervention, Cognitive Performance, and Brain Structure: Results from the Physical Influences on Brain in Aging (PHIBRA) Study
Source: Front Aging Neurosci. 2017 Jan 18;8:336. doi: 10.3389/fnagi.2016.00336 (PMC5241294; doi:10.3389/fnagi.2016.00336)
Supplement: Supplementary file 1 [file Presentation1.PDF]

Supplementary Material

Physical Influences on Brain in Aging (PHIBRA): Aerobic exercise intervention, cognitive performance, and brain structure

Lars S Jonasson*, Lars Nyberg, Arthur F Kramer, Anders Lundquist, Katrine Riklund, Carl-Johan Boraxbekk

*** Correspondence:** Lars Jonasson: lars.jonasson@umu.se

1 Supplementary 1

A five factor solution with a 'Cognitive score' latent variable with loadings from EM, PS, UPD, and EF latent variables showed best fit, $\chi 2(50, N = 118) = 75.199$, $p < .012$, RMSEA = .065, CFI = .946, AIC = 3652.311, SRMR = 0.079 (Supplementary Figure 1). When TS tasks were added to the model there were problems with the covariance matrices, thus they were dropped from further analysis. A four factor solution without 'Cognitive score', including EM, PS, UPD, and EF, also showed good fit, $\chi 2(48, N = 118) = 73.942$, $p < .009$, RMSEA = .068, CFI = .944, AIC = 3654.891 (Supplementary Figure 2).

For the reasoning tasks, the fit for the two factor solution was better, $\chi 2(4, N = 121) = 11.971$, p < . 0.018, RMSEA = 0.128, CFI = 0.962, AIC = 1563.734, SRMR = 0.044 (Supplementary Figure 3), than for the single factor solution, $\chi 2(4, N = 121) = 32.512$, p < . 0.001, RMSEA = 0.213, CFI = 0.870, AIC = 1582.275, SRMR = 0.066.

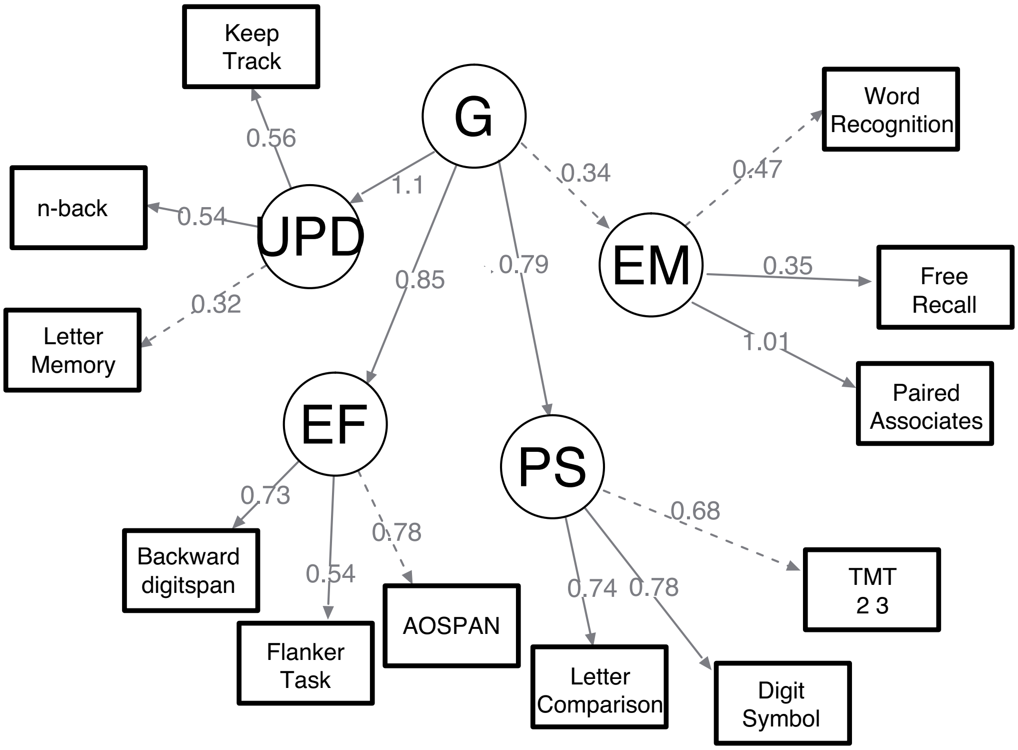

Supplementary Figure 1. Five factor solution SEM using the maximum likelihood estimation with robust standard errors. All factor loadings were significant at $p < .05$. and fit indices indicated a good fit, $\chi 2(50, N = 118) = 75.199$, $p < .012$, RMSEA = .065, CFI = .946, AIC = 3652.311, SRMR = 0.079. UPD = updating, EM = episodic memory, PS = processing speed, EF = executive function, G = 'Cognitive score', AOSPAN = Automated Operation Span, TMT = Trail Making Task.

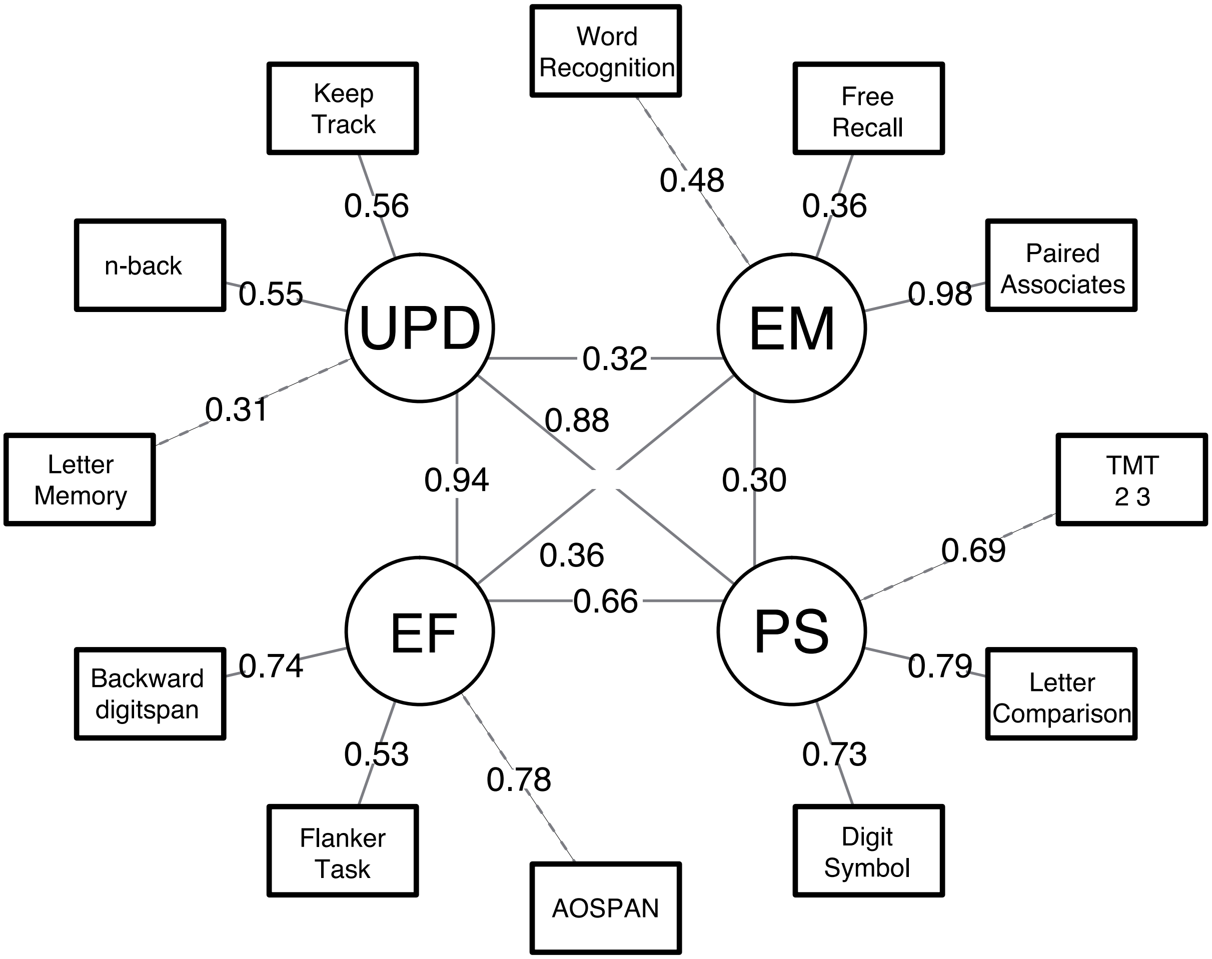

Supplementary Figure 2. Four factor solution SEM using the maximum likelihood estimation with robust standard errors. All factor loadings were significant at $p < .05$. and fit indices indicated a good fit, $\chi2$ (52, N = 121) = 74.188, $p < .009$, RMSEA = .067, CFI = .928, AIC = 3736.168. UPD = updating, EM = episodic memory, PS = processing speed, EF = executive function, AOSPAN = Automated Operation Span, TMT = Trail Making Task.

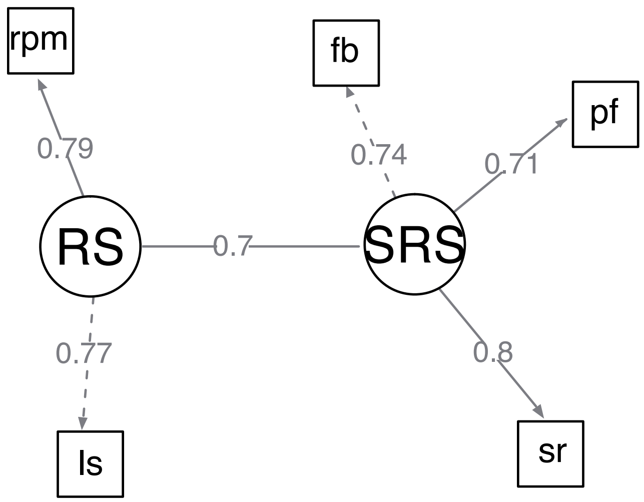

Supplementary Figure 3. Two factor solution SEM using the maximum likelihood estimation with robust standard errors. All factor loadings were significant at p < .05, $\chi 2(4, N = 121) = 11.971$, p < . 0.018, RMSEA = 0.128, CFI = 0.962, AIC = 1563.734, SRMR = 0.044. ls = Letter sets, rpm = Ravens Progressive Matrices, fb = Form boards, pf = Paperfolding, sr = Spatial relations, RS = Reasoning, SRS = Visuospatial ability.

Supplementary Table 1. Neuropsychological test battery.

	Aerobic						Control									
	Baseline			6-months			Baseline			6-months			Change			
Statistic	N	Mean	SD	N	Mean	SD	N	Mean	SD	N	Mean	SD	δ_{RM}	p[a]	δ_{RM}	p[a]
**Episodic Memory (z)**	29	-0.17	0.50	29	0.08	0.86	29	0.22	0.87	29	0.17	1.02	0.41	#	-0.08	
Word Recognition (%)	29	0.74	0.10	29	0.77	0.13	29	0.77	0.10	28	0.75	0.13	0.23		-0.23	
Free Recall (C)	29	5.59	1.84	29	6.35	2.99	29	6.35	2.02	29	6.52	2.79	0.30		0.07	
Paired Associates (C)	29	9.14	2.67	29	9.07	3.61	27	11.04	4.74	29	10.72	4.17	-0.02		-0.12	
**Processing Speed (z)**	29	0.08	0.61	29	0.37	0.47	29	-0.12	0.99	29	0.05	1.04	0.76	***	0.40	*
TMT 2-3 (s)	29	89.47	26.86	29	78.70	17.90	29	95.30	38.09	29	90.89	37.25	-0.67	**	-0.23	
Digit Symbol (ms)	29	2632	306	29	2516	312	29	2805	467	29	2620	444	-0.54	**	-0.65	**
Letter Comparison (ms)	29	884	113	29	851	79.68	29	884	156	29	895	200	-0.33		0.12	
**Updating (z)**	29	-0.09	0.49	29	0.10	0.51	29	0.06	0.61	29	0.12	0.72	0.42	*	0.14	
Letter Memory (%)	29	0.28	0.20	29	0.23	0.19	29	0.29	0.22	28	0.28	0.17	-0.24		-0.05	
2-back (%)	29	0.69	0.21	29	0.76	0.18	27	0.73	0.20	28	0.75	0.21	0.54	**	0.14	
Keep Track (C)	29	13.35	2.48	28	13.43	2.25	29	12.93	3.37	29	12.83	3.58	0.03		-0.03	
**Task-switching (z)**	29	-0.10	0.59	29	0.14	0.56	29	0.07	0.63	29	0.15	0.53	0.30		0.10	
TMT 4 (cost s)	29	18.17	28.47	29	10.08	19.52	29	7.62	25.12	29	0.24	21.08	-0.37	#	-0.22	
Odd Even (cost %)	29	0.05	0.06	29	0.03	0.06	28	0.04	0.08	27	0.02	0.05	-0.27		-0.20	
Local Global (cost %)	28	0.02	0.08	29	0.01	0.10	27	0.02	0.08	29	0.05	0.07	-0.07		0.39	#
**Executive Function (z)**	29	0.07	0.71	29	0.50	0.76	29	-0.06	0.89	29	0.16	0.91	0.79	***	0.45	**
Automated Operation Span (C)	28	25.14	8.98	29	30.41	9.37	26	24.46	11.72	29	26.21	12.52	0.95	**	0.20	#
Flanker Task (cost ms)	29	218	139	29	164	125	28	272	177	29	228	185	-0.36	#	-0.24	
Backward Digit span (C)	29	4.07	1.56	29	4.69	1.58	29	4.17	1.28	29	4.38	1.35	0.44	*	0.19	
**Reasoning (z)**	29	0.10	0.81	29	-0.15	0.94	29	-0.07	0.99	29	-0.04	0.96	-0.40		0.07	
Letter Sets (C)	29	5.95	2.83	29	5.17	2.96	29	5.18	3.03	29	5.12	3.21	-0.35		-0.03	
Ravens (C)	29	3.06	2.13	29	2.51	2.53	29	2.88	2.72	29	3.08	2.59	-0.30	#	0.11	
**Visuospatial Ability (z)**	29	0.01	0.83	29	0.26	0.86	29	0.03	0.87	29	0.05	0.96	0.56	*	0.03	
Form Boards (C)	29	4.97	3.28	28	6.04	2.94	29	4.62	2.81	29	5.93	3.29	0.60	*	0.65	***
Paper Folding (C)	29	2.92	2.02	29	3.34	2.40	28	2.99	2.41	29	2.50	2.63	0.23		-0.18	
Spatial Relations (C)	29	3.26	3.71	29	3.93	3.32	28	3.69	3.64	29	3.21	4.00	0.24		-0.21	
**Cognitive Score**	29	-0.03	0.42	29	0.26	0.50	29	0.02	0.62	29	0.13	0.67	1.02	***	0.43	**

Note. Cognitive constructs and 'Cognitive score' are reported as z scores, where a positive number denotes an improvement. Task scores are reported in outcome metrics. For completion, all constructs and tasks will be reported in uncorrected group-specific repeated measures analysis of variance. In addition, effect size δ_{RM} estimates for each test and group were calculated. The formula provided by (Morris & DeShon, 2002), equation 8, was used to calculate effect sizes as it takes into account the test correlation between time points when calculating the effect size for repeated measures. [a]p value

from group specific repeated measures analysis of variance. #$p < 0.1$, *$p < 0.05$, **$p < 0.01$, ***$p < 0.001$.
